# Sociosanitary Legal and Economic Aspects in Nursing Homes for the Elderly in Spain

**DOI:** 10.3390/ijerph20064928

**Published:** 2023-03-10

**Authors:** Cristina Vilaplana-Prieto, Carmelo S. Gómez Martínez, Paloma Echevarría Pérez, Isabel Legaz

**Affiliations:** 1 Department of Fundamentals of Economic Analysis, Faculty of Economics and Business, University of Murcia, 30120 Murcia, Spain; 2 Faculty of Nursing, Universidad Católica de Murcia (UCAM), 30107 Murcia, Spain; 3 Department of Legal and Forensic Medicine, Faculty of Medicine, Biomedical Research Institute (IMIB), Regional Campus of International Excellence Campus Mare Nostrum, University of Murcia, 30100 Murcia, Spain

**Keywords:** autonomy, elderly, legal medicine, residential homes, welfare state, nursing homes

## Abstract

Aim: The study aimed to identify and compare aspects of the different Spanish regulations on the minimum conditions that nursing homes must meet and to compare whether these requirements significantly affect the price of a nursing home place in each region. Methods: We analyzed and compared the 17 regional regulations that must be met by nursing homes in terms of equipment and social and healthcare staff and combined this information with regional information concerning the price and coverage of public and subsidized places in nursing homes. Results: The study revealed significant regional inequality in physical facilities and human resources. However, the number of regulatory measures referring to the mandatory availability of physical space or specific material resources was not positively correlated with an increase in the price of a place in a public or subsidized nursing home. Conclusions: No unified regulations throughout Spain regulate the aspects that residential centers must comply with. There is a need to move towards a person-centered approach, providing an environment as close to home as possible. The regulation of minimum standards to be met by all nursing homes at the national level should not significantly impact prices.

## 1. Introduction

Spain has one of the highest life expectancies in the world [1,2,3,4,5,6,7,8], which is why the aging of the population represents one of the most critical challenges we face in all social, health, economic, and cultural spheres [9,10]. The elderly population currently constitutes a majority group in social and healthcare care, both in Spain and the rest of the countries around it, and is fundamentally characterized by its progressive vulnerability [11].

The Spanish Constitution established that autonomous communities (denominations for regions) can assume competencies in “social care” [12]. By this precept, all the autonomous communities assumed exclusive competence for social services in their respective statutes of autonomy and reiterated this competence in the latest statutory reforms. Consequently, in Spain, social services are a part of the welfare state that varies geographically between societies and provinces, and even municipalities within the same province [12,13,14].

The design of the Spanish territorial government as a decentralized model of territorial organization generates significant dysfunctions and inequalities [15,16,17,18]. The coexistence of 17 regulations (corresponding to the 17 autonomous communities) for social services, health, and education, with different levels of protection and funding [15], generates a very asymmetrical context [19,20,21].

Moreover, a non-neglectable proportion of older Spanish people (298,077 in 2020) spend their final years in nursing homes and day centers [22,23,24]. Additionally, the pandemic caused by SARS-CoV-2 highlighted various problems, ethical–legal dilemmas, deep inequalities among the most vulnerable and defenseless in our population, and inconsistencies between health and social services in nursing homes or day centers [25,26,27]. As a result, their responsiveness, short- and long-term feasibility, equitable access, and ability to smoothly and dynamically protect this vulnerable sector of society in the face of political, social, and health changes were questioned. Nursing homes and daycare centers are legislated social institutions [28] in a national framework; however, they undergo changes depending on the social, political, and geographical region in which they are located [29,30]. In this context, they envisage a bleak future if the appropriate corrective measures are not taken, given the aging of the population, emerging loneliness, changes in family and community roles [31,32], as well as how the re-emergence of new pandemics could endanger the lives of vulnerable sectors of the population, whom we have a moral, legal, and social obligation to protect [33,34].

We believe that the present moment is an excellent time to carry out this analysis, given that, in 2022, the Ministry of Social Rights and Agenda 2030 approved the agreement on standard criteria for accreditation and quality of the centers and services of the System for Autonomy and Care for Dependency [35,36]. One of the innovations of this agreement was the creation of cohabitation units within the nursing homes that group together a maximum of 15 people so that they live in an environment that is as close to home as possible. This novelty is a challenge because the current regulatory framework, as will be seen further on, reveals a high degree of heterogeneity. On the other hand, in this new scenario, the question arises as to whether further regulation of the characteristics of nursing homes at the national level could significantly impact their prices. Although it is undoubtedly impossible to know what the situation would be in a counterfactual scenario (i.e., if all the autonomous communities currently shared the same regulatory framework), we attempted to carry out a simple estimation exercise by analyzing whether a strict regulatory framework had a significant impact on the price (and number) of residential care homes. The empirical literature contained little evidence of these variables’ relationship, so our work constituted a novel contribution. Mukamel et al. [37] analyzed US residential centers’ cost functions and reported that homes in states with more stringent regulatory requirements faced higher costs, ceteris paribus. Considering that these regulations were intended to set minimum standards, typically related to quality and safety, Clement et al. [38] and Herr and Hottenrott [39], both for the US and Germany, found that homes offering excessively low prices were more likely to have an insufficient quality of services. As Heger et al. [40] highlighted, the counterpart is that a higher price (associated with higher quality) also means a higher co-payment for the user. Concerning the availability of human resources, there is no conclusive evidence on the relationship between quality of care and quantity of qualified personnel. While Grabowski [41] observed a positive correlation between the two variables, other authors pointed to the inadequate use of sedative drugs [42].

Consequently, our main objectives are the following ones. First, to understand and compare aspects of the different regulations in Spain governing the minimum conditions that must be met by nursing home facilities in order to establish points for improvement, promote equity and stimulate the possible transition towards new residential models that provide person-centered care that favors the well-being and autonomy of the elderly. Second, analyze the relationship between the degree of regulation of nursing homes, the price per place, and the coverage rate.

## 2. Material and Methods

### 2.1. The Spanish System for Autonomy and Care for Dependency

The text of the Spanish Constitution, in articles 49 and 50, refers to the care of disabled and older adults and a system of social services promoted by the public authorities. In 1978, the fundamental elements of this welfare state model focused on health protection and the social security system for all citizens. However, the transformation of social structures led to the increasing importance of social services for the dependent population, organized mainly by autonomous communities, in special collaboration with private, non-profit organizations, as the new pillar of the welfare system (together with healthcare, education, and pensions).

A new citizenship right, known as the “fourth pillar of the welfare system,” was established by Law 39/2006, of 14 December, on the Promotion of Personal Autonomy and Cared for Dependent Persons (LAPAD) [43]. This law recognized the universal nature of benefits and the right to access them equally for all elderly or disabled people who need help carrying out essential daily activities. The system is known as the System for Autonomy and Care for Dependency; (Sistema para la Autonomía y Atención a la Dependencia, or SAAD, using the Spanish acronym). The principles on which this law was based were to guarantee the primary conditions of well-being and to provide the necessary levels of protection on a universal basis for the entire population.

Prior to the SAAD, the needs of the elderly and dependent persons were addressed essentially by the autonomous regions and at the local level (in the context of the Concerted Plan on Basic Social Services Provisions) and at the state level (through the Action Plans for the Disabled and the Elderly). On the other hand, social security assumed responsibility for some elements of care, both in the care of the elderly and in situations linked to disability: (i) severe disability benefits, (ii) non-contributory disability pensions, (iii) family benefits for dependent children, and (iv) social service benefits in the re-education and rehabilitation of people with disabilities.

The Dependency Assessment Scale allows the determination of situations of moderate dependency, severe dependency, and significant dependency. Moderate dependency is when the person needs help carrying out several basic activities of daily living at least once a day or has intermittent or limited support for personal autonomy. Severe dependency is when the person needs help to perform several basic activities of daily living two or three times a day but does not require the permanent support of a carer or has extensive support needs for personal autonomy. High dependency occurs when the person needs help to perform several basic activities of daily living several times a day and, because of a total loss of physical, mental, intellectual, or sensory autonomy, needs another person’s permanent support or extensive support needs for personal autonomy.

The Dependency Assessment Scale [44] evaluates 47 tasks grouped into the following ten activities of daily living: eating and drinking, control of physical needs, bathing, and basic personal hygiene, other personal care, dressing and undressing, maintaining one’s health, mobility, moving outside the home, and housework. Each activity of daily living is assigned a different weight, and there is a different scale for individuals with mental illness or cognitive disability. Additionally, the evaluation considers the degree of supervision required to perform each task. The final score is the sum of the weights of the activities of daily living for which the individual has difficulty, multiplied by the degree of supervision required. The degree of dependency is determined as the result of the sum: not eligible (less than 25 points), moderate dependency (25 to 49 points), severe dependency (50 to 74 points), and major dependency (above 74 points). Spain’s Royal Decree 504/2007, of 20 April, approved the dependency rating scale established by Act 39/2006, of 14 December, Promoción de la Autonomía Personal y Atención a las Personas en Situación de Dependencia.

Persons certified as dependent are entitled to receive care and attention through services appropriate to their degree and level of dependency (individual care program or ICP). An ICP determines the services or benefits that best meet the applicant’s needs. This program is established with the beneficiary’s participation through consultation and opinion-seeking and, where appropriate, with his/her family.

The autonomous communities assumed the responsibility for providing the benefits and services established by the LAPAD within the framework of the so-called Social Services Network of the autonomous communities. These competencies include not only the provision of services to dependent persons but also the granting of certain economic benefits.

The catalog of services includes services for preventing dependency and promoting personal autonomy, telecare, home care, day and night center service, and nursing homes.

When the competent administrations cannot provide these services, the dependent person is entitled to economic benefits. Service-linked financial benefit, only awarded when care is not possible through a public care service, the financial benefit for care in the family environment and support for non-professional carers, and allowance for personal assistance to facilitate the beneficiary’s access to education and employment is provided.

### 2.2. Spanish Legal Regulations of the Nursing Homes

In this study, we analyzed and compared 17 Spanish legal regulations on the minimum requirements to be met by nursing homes in Spanish autonomous communities [45] (Table 1). All regulations of Spanish systems were considered in this study.

### 2.3. Variables Analyzed in the Regulations for Nursing Homes

A total of 13 requirements of nursing homes were analyzed and compared for the 17 Spanish autonomous communities (Table 2).

### 2.4. Statistical Analysis

In the statistical analysis of the paper, we used information from the following sources: (1) public price of a place in a nursing home (EUR /year), concerted price of a place in a nursing home (EUR /year), and coverage rate of public places and concerted places for all the Autonomous Communities in 2020, from the report “Servicios Sociales dirigidos a personas mayores 2020”, available in open access on the website of IMSERO (Instituto de Mayores y Servicios Sociales, Madrid, Spain) [54]; (2) gross domestic product (GDP) per capita for each autonomous community in 2020 from the Spanish Regional Accounts (available on the website of the Spanish National Statistics Institute (INE) [62].

The coverage rate was the number of residential places per 100 inhabitants aged 65 and over. Ordinary least squares (OLS) regressions were estimated for the public or subsidized price and the coverage or subsidized rate as a function of the regulations introduced in each community (and also of the price, in the case of the regressions for the coverage rate). The software used was STATA 15.0 (StataCorp. 2015. Stata Statistical Software: Release 15. College Station, TX, USA: StataCorp LP)

## 3. Results

### 3.1. Comparative Analysis of Nursing Homes Requirements

The analysis of the minimum dining area showed that the regulations of Aragón, Castilla y León, Cataluña, Comunidad Valenciana, Extremadura, Región de Murcia, Navarra, Basque Country, and La Rioja did not specify any requirement. Regulation for Madrid established a minimum of 1 m^2^ per user. The remaining regions (Andalusia, Asturias, the Balearic Islands, and the Canary Islands) set a minimum surface area of 2 m^2^ per user (Table 3).

For kitchens, Cantabria, Catalonia, and La Rioja required a minimum surface area per resident of more than 0.50 m^2^ per user, while Andalusia, Castile-La Mancha, and Valencia set the minimum surface area regardless of the number of users. The remaining autonomous communities did not specify any requirement. Most autonomous communities established the minimum surface area of living rooms. Andalusia, the Canary Islands, and Castilla y Leon required a minimum surface area of 30 m^2^, but this feature was not included in the Region of Murcia, the Valencian Community, and the Basque Country. Andalusia and the Region of Murcia were the only regions that regulated the minimum surface area of visiting rooms (15 m^2^ and 12 m^2^, respectively).

As for the availability of a chapel and/or mortuary room, the Region of Murcia was the only one that specified the mandatory nature of this facility in those municipalities where there was no public service. Other autonomous communities (Navarre, Asturias, the Balearic Islands, Castile, Leon, Catalonia, and the Valencian Community) mentioned these facilities in their regulations, but voluntarily. Details of the regulations in each autonomous community are given in Table 3.

Concerning outdoor recreational areas (Table 3), they were mentioned in the regulations of Andalusia, the Balearic Islands, the Canary Islands, Castile-La Mancha, and the Valencian Community. As for the possibility of customizing residents’ rooms, this feature was only included in Asturias, the Balearic Islands, Castile, Leon, Catalonia, and Galicia.

Hairdressing areas were only regulated in Cantabria, Castilla-La Mancha, and the Valencian Community. The regulations of Cantabria established a hairdressing area for residential centers with more than 50 beds and those of Castilla-La Mancha for centers with more than 80 beds. On the other hand, the Valencian Community’s regulations specified these areas’ provisions but without establishing criteria for various users.

Concerning lifts and freight lifts, the regulations of the Region of Murcia established that centers with more than one floor must be equipped with a lift with minimum dimensions of 2.10 by 1.10 m. Other autonomous communities such as Andalusia, Castilla-La Mancha, the Balearic Islands, and La Rioja set the number of lifts according to the number of residents. The remaining autonomous communities did not specify when a lift was required. Finally, the autonomous communities with an annual program of sociocultural activities were the Valencia Community, the Basque Country, and the Region of Murcia.

### 3.2. Regulation of Health Staff Requirements in the Nursing Homes

The ratio of doctors (number of doctors to number of residents) was only included in the regulations of Castilla-La Mancha, Castilla y León, Comunidad Valenciana, Extremadura, Galicia, Región de Murcia, Basque Country, and La Rioja. The rest of the autonomous communities did not specify a minimum requirement (Table 4).

In Castilla la Mancha, nursing homes with more than 45 beds required a doctor 5 h per week, which was increased by 1 h/per week for every ten residents or a fraction thereof, up to residences with 119 beds. Those with 120 beds or more were required to have a doctor available for 17 h/per week, increasing by 8 h per week for every 20 users or a fraction thereof. In Castilla y León, residences with up to 25 users required a minimum service of 1 doctor per day, increasing by another hour/week for each additional 25 users or a fraction thereof.

In the Valencian Community, a minimum weekly service of 5 h was established for every ten places or a fraction thereof in centers with less than 100 places. In centers with more than 100 places, 10 h/week for every 25 places or a fraction thereof.

In Extremadura, the availability of a doctor was established (without specifying hours). In Galician centers with less than 40 assisted places, a doctor was required 24 h per day. As for nursing homes in Murcia, when there were up to 40 users, a minimum of one hour per day was required. In addition, for each additional 20 users, the doctor provided a service of one hour more per day.

The Basque Country established the ratio of doctors according to the degree of dependency of each nursing home’s residents. In La Rioja, the ratio of doctors was established according to the occupancy level of its residential centers. A doctor was available for 20 h/week for moderate dependents. It was increased to 40 h/week; for severely dependent persons, it was further increased to 80 h/week.

On the other hand, the autonomous communities that regulated the number of nurses (about the number of residents) were Asturias, Castilla-La Mancha, Castilla y León, Comunidad Valenciana, Extremadura, Galicia, Región de Murcia, Navarra, Basque Country, and La Rioja. The rest of the autonomous communities did not regulate the nursing ratio in residential centers (Table 4).

Asturias set a ratio of 0.025 nurses per user considered severely dependent, and 0.035 nurses per user classified as highly dependent were established. Castilla la Mancha established 20 h/week for mini nursing homes and 40 h/week for medium and large nursing homes with more than 80 beds. There was 40 h of nursing service per week, which was increased by 20 h per week for every 40 users or a fraction thereof.

In Castilla y León, residences with more than 25 users required a minimum service of 2 h/day, with an increase of 1 h/day for every eight users or a fraction thereof. In the Valencian Community, a minimum of eight hours/week was required for every 19 users or fraction proportionally, but high-dependency nursing homes required a permanent nurse. In Galicia, a permanent nurse was required.

In Extremadura, only one nurse for every 25 residents was required. The Region of Murcia required a nurse with a minimum service of 1 h/day for up to 40 users. In addition, for every 20 users or a fraction thereof, an additional hour per day was provided, while, in Navarre, a permanent nurse was in each nursing home.

In the Basque Country, it depended on the degree of dependency of the residents (non-dependent and moderate dependents: 0.027 h/user; severe dependents and significant dependents: 0.032 h/user). Finally, In La Rioja, a nurse was available in the following proportion: 60 h/week for non-dependents, 80 h/week for moderate dependents, 100 h/week for severe dependents, and 160 h/week for heavy dependents.

### 3.3. Effect of Regulation on Price and Coverage

Detailed information on prices, coverage rate, and coverage index is shown in Table 5.

**Figure 1:**
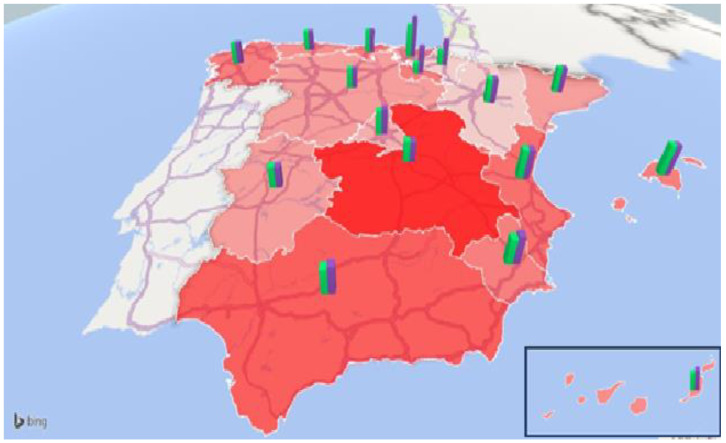
Relation between degree of nursing home regulation (red areas), price of a public residential home (green bricks), and price of subsidized places in nursing homes 2020 (purple bricks). Source: own work using information from Table 3 and Table 4 and [63]. Detailed prices (EUR/year) are shown in Table 6.

The high degree of regional dispersion is noteworthy since the price of the public (subsidized) places ranged between 10,423.57 EUR/year and 27,342.67 EUR /year (15,591.57 EUR/year and 34,721.45 EUR/year), which meant a difference of 16,919.10 EUR/year in the public places and 19,129.88 EUR/year in the subsidized places. The aim of displaying the degree of regional regulation and public/subsidized prices together was to attempt to perceive whether there was any relationship between these variables. A priori, one could conceive that a higher degree of regulation may translate into higher costs (i.e., construction costs to comply with the minimum required surface areas and labor costs to hire the necessary personnel). However, the picture in Figure 1 indicates this was not the case. Castilla La Mancha was the region with the highest number of regulatory measures, and yet, the price per public (subsidized) place was 10,977.01 EUR/year (18,671.71 EUR/year) lower than the price in the Basque Country, whereas the number of regulatory measures was much lower (only six). In addition, the difference between public and subsidized prices was maximum in La Rioja (12,513.51 EUR/month), Navarra (8787.36 EUR/month), and Basque Country (7378.78 EUR/month).

**Figure 2:**
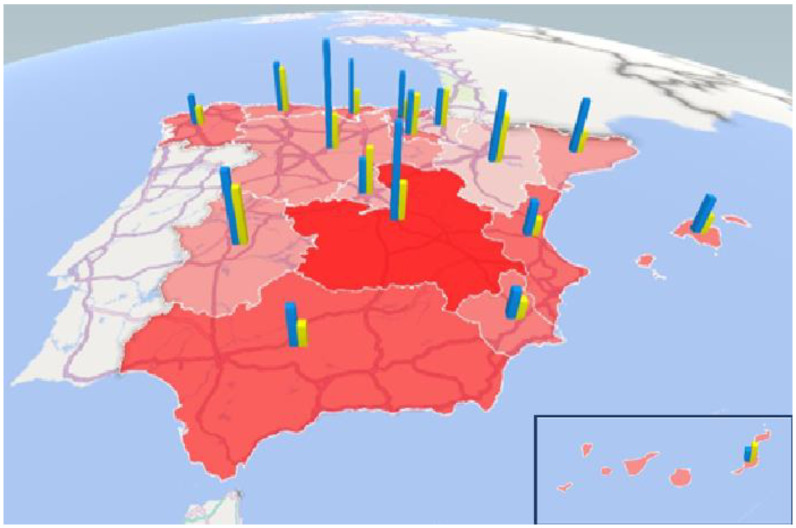
Relation between degree of nursing home regulation (red areas), coverage of public residential homes (blue bricks), and coverage of subsidized places in nursing homes 2020 (yellow bricks). Source: own work using information from Table 3 and Table 4 and social services aimed at the elderly in Spain. December 2020—Institute for the Elderly and Social Services [64]. Coverage rate: number of beds placed per 100 persons aged 65 and over.

Given the extent and density of the abovementioned regulations, we tried to summarize them in Figure 1 and Figure 2. Figure 1 shows the regional situation of nursing homes in 2020. The areas shaded in red indicate the degree of regional regulation. For this purpose, 13 binary variables were defined, taking the value one if, for each region, there was a specific regulation in the following aspects: minimum surface area of dining rooms, kitchens, and living rooms; mandatory hairdressing area, visiting room, wake chapel/mortuary, elevator, and cranes; sociocultural activities program; room customization; the ratio of nurses and ratio of physicians. All these variables were then summed up in a single indicator that summarized the degree of regulation in logistics and human resources of the residences for each region. The greater the intensity of the red color (for example, Castilla La Mancha), the greater the regulation, and the lesser the intensity (for example, Aragón and Navarra), the greater the degree of freedom. On the other hand, the green and purple bricks represent the price (EUR/year) of a place in a public and subsidized nursing home in 2020. The difference between both was in the management, since public places correspond to social services, while subsidized ones correspond to private residences, where several places are reserved for public social services.

In Figure 2, we combined the information on the number of regulatory measures and nursing home places in 2020. Since the aging of the population may influence the number of places at a regional level, we chose to use the coverage rate as an indicator, i.e., the number of nursing home places (public or subsidized) per 100 persons aged 65 and over. Only five communities (Aragon, Asturias, Castilla y Leon, Castilla La Mancha, and Extremadura) exceeded the WHO recommended threshold of five residential places per 100 persons aged 65 and over. We observed that there were communities with high regulation (Castilla La Mancha with thirteen regulatory measures) and low regulation (Extremadura with six) but with high coverage of subsidized places. At the other extreme, in Aragon (with only two regulatory measures), the coverage of subsidized places was the highest in Spain. In the case of public places, no correlation (positive or negative) between the two variables was observed either.

Table 6 shows the correlation matrix between price (public and subsidized), coverage rate (public and subsidized), GDP per capita (GDPpc), and some facilities and human resources requirements. We observed a significant and negative relationship between the number of requirements (facilities and human resources) and the price (public and subsidized) and only a significance of 10% between GDPpc and the public market price. The correlation between many facility requirements and the (public or subsidized) coverage rate was only significant at 10%. No significant relationship was observed between price and coverage ratio.

Although an overly excessive and coercive regulatory framework can lead to higher costs for residential placements, it should also be kept in mind that better design of residences can provide better compensation for residents’ physical and sensory weaknesses, giving them greater control over their environment, even when a cognitive disability is present [65,66].

**Figure 3:**
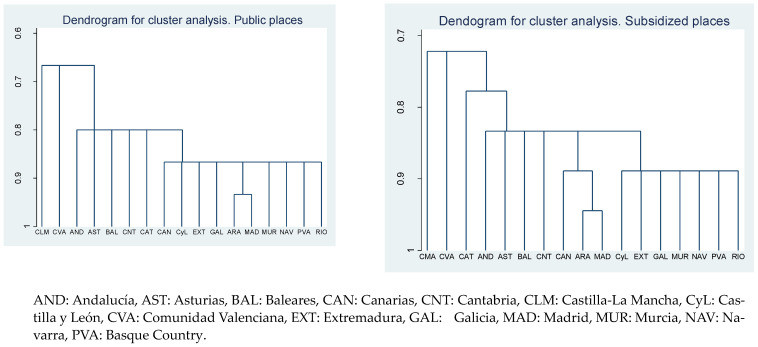
Dendrogram for cluster analysis.

Figure 3 shows two dendrograms for public and subsidized places using as explanatory variables: prices, coverage ratios, binary variables related to all requirements (facilities and human resources), and GDPpc. In both dendrograms, we highlighted: (i) Castilla León, Extremadura, Galicia, Murcia, Navarra, Basque Country, and La Rioja were in the same group; (ii) within the previous group, Aragón and Madrid formed a subgroup of their own; (iii) Andalucía, Asturias, Baleares, and Cantabria formed a group with an intermediate level of similarity; (iv) Castilla La Mancha and Comunidad Valenciana were not integrated with any of the previous groups.

A simple econometric model was estimated to explore further these regulatory measures’ effects (Table 6). We estimated by ordinary least squares two regressions for prices and another two regressions for coverage rates:

(1) $${PP}_{i}={R\prime }_{i}\alpha_{o}+\alpha_{1}{\mathrm{TR\_}F}_{i}+\alpha_{2}{\mathrm{TR\_}HR}_{i}+\alpha_{3}{GDPpc}_{i}+\alpha_{4}{\mathrm{CR\_}PP}_{i}+\epsilon_{i}$$

(2) $${SP}_{i}={R\prime }_{i}\alpha_{o}+\alpha_{1}{\mathrm{TR\_}F}_{i}+\alpha_{2}{\mathrm{TR\_}HR}_{i}+\alpha_{3}{GDPpc}_{i}+\alpha_{4}{\mathrm{CR\_}SP}_{i}+\zeta_{i}$$

(3) $${\mathrm{CR\_}PP}_{i}={R\prime }_{i}\alpha_{o}+\alpha_{1}{\mathrm{TR\_}F}_{i}+\alpha_{2}{\mathrm{TR\_}HR}_{i}+\alpha_{3}{GDPpc}_{i}+\alpha_{4}{PP}_{i}+\varepsilon_{i}$$

(4) $${\mathrm{CR\_}SP}_{i}={R\prime }_{i}\alpha_{o}+\alpha_{1}{\mathrm{TR\_}F}_{i}+\alpha_{2}{\mathrm{TR\_}HR}_{i}+\alpha_{3}{GDPpc}_{i}+\alpha_{4}{SP}_{i}+\xi_{i}$$

where ${PP}_{i}$ and ${SP}_{i}$ denote the public price and subsidized price (thousand EUR/year) in autonomous community *i*; ${R\prime }_{i}$ represents a vector of binary variables for residential requirements (binary variables for the following variables (dining rooms (required minimum area), kitchens (required minimum area), living room (required minimum area), hairdressing area (availability), visiting room (availability and required minimum area), chapel and/or mortuary (availability), sanitary mobilization cranes (availability), lift (number and availability), room customization, outdoor recreation area (availability), sociocultural activities program), ratio of nurses and ratio of doctors); ${\mathrm{TR\_}F}_{i}$ denotes the total number of requirements related to facilities’ characteristics and ${\mathrm{TR\_}HR}_{i}$ the total number of requirements related to human resources; ${\mathrm{CR\_}PP}_{i}$ and ${\mathrm{CR\_}SP}_{i}$ indicate the coverage rate for public prices and subsidized places; ${GDPpc}_{i}$ is the per capita gross domestic product for autonomous community *i* (thousand euros), and finally, $\epsilon_{i}$, $\zeta_{i}$, $\varepsilon_{i}$ and $\xi_{i}$ denote error terms.

In addition, we proceeded with a progressive inclusion of the explanatory variables. In the regressions for price per place, we first included all the requirements-related variables, then GDPpc and the coverage rate. In the regressions for the coverage rate, we first included all the variables related to requirements, then GDPpc, and finally, the price per place.

Table 7 and Table 8 show the estimated coefficients for the regressions for the price per square and the coverage ratio, respectively. If we consider the sign of the estimated coefficients, we observe that the total number of regulatory measures (for facilities and human resources) was negatively associated with the (public or subsidized) price. Concerning requirements for facilities, the regulation on dining rooms, kitchens, living rooms, or chapels/mortuaries was negatively associated with the price of the public (or subsidized). The possibility of room customization and the availability of outdoor recreation areas were not significant variables. On the contrary, the lift or hairdressing area requirement positively correlated with the price. As for human resources, requirements for nurses and doctors were negatively associated with the public or subsidized price. Comparing the magnitude of both types of requirements, those for facilities exerted a more intense effect on the price of subsidized places, whereas those related to human resources exerted a greater effect on the price of public places. The effect of GDPpc was only significant for the price of public places, although with a small magnitude. The coverage rate (for public or subsidized places) was insignificant in any regression.

Regarding the results for the regressions corresponding to the coverage of residential places, we observed that the number of regulatory measures was positively associated with the availability of places (public or subsidized). In particular, the variables living room, hairdressing, visiting room, and sociocultural activities reflected these positive associations. A total number of facilities-related requirements exerted a significant and positive effect on coverage rate (public or subsidized), which was more intense in the former ones. In contrast, neither human resource requirements nor GDPpc or price were significant variables.

## 4. Discussion

The aim of this study was, firstly, to compare the different regulations in Spain governing the minimum conditions that must be met by nursing home facilities, and secondly, to establish points for improvement, promote equity in nursing homes, and stimulate the possible transition to new residential models that provide person-centered care favoring the well-being and autonomy of the elderly.

The organization of health care for the elderly varies greatly between countries. In the United States, the Medicare program provides health coverage to those over 65. There are also a variety of private insurance plans that offer additional coverage for seniors. In addition, many states have programs to assist seniors needing long-term care [67].

In the United Kingdom, the National Health Service (NHS) provides care for all citizens, regardless of age. In addition, the NHS provides free hospital care and medical services to those over 60 [68]. In France, the national government administers the public health system funded by taxes. Those over the age of 65 are entitled to free health care services. In addition, the government provides additional benefits, such as free prescription drugs and home care services, to those in need [69]. In Canada, the health care system is funded by both the federal and provincial governments. Seniors are entitled to various services and benefits, such as subsidized prescription drugs and home care services [70]. In Germany, the public health system is funded by taxes and provides universal coverage for all citizens. Those over 65 are entitled to additional benefits, such as free prescription drugs and home care [71].

Our data showed that the minimum conditions that regulate nursing homes were neither unified nor homogeneous for the entire Spanish territory since each autonomous community established, according to its regulations, the requirements that their nursing homes must meet.

Our result of a non-positive correlation between price (or coverage) and degree of regulation was essential given that in 2022, the Ministry of Social Rights and Agenda 2030 [71] approved the agreement on standard criteria for accreditation and quality of the centers and services of the System for Autonomy and Care for Dependency. One of the novelties of this agreement was that it introduced the idea of “cohabitation units” within the nursing home itself, in which a maximum of 15 people cohabit. These spaces resemble a home in their architecture, decoration, and furniture, as well as in the routines and schedules adapted to the preferences and habits of the people living in them, favoring their participation, autonomy, comfort, stimulation, orientation, and well-being. They comprise a shared space, which includes an area for preparing meals, a dining room and living room for residents and their relatives, and the bedrooms of the people living in the unit. It is advisable that they also have access to an outdoor area, such as a terrace or garden. These units are delimited, identified, and differentiated from other cohabitation units of the same center and define the spatial structure of nursing homes with the dimension and atmosphere of a home. Additionally, each resident will have a personal care and support plan, which will include the preferences and wishes of the person’s will concerning how they want to live, as well as the issues that are significant for them.

Although we observed a significant and negative relationship between facility requirements and human resources on the public and subsidized price, we recognize that one of the limitations of this work was that we could not control the location of the residences. This aspect becomes particularly relevant in the agreement on standard criteria for accreditation and quality of the centers and services of the System for Autonomy and Care for Dependency, which stipulated a limit to the number of places depending on the location of the residence (rural, intermediate, or urban area). This can influence economies of scale and the greater or lesser ease of hiring social and healthcare professionals.

The residential models in Spain are mainly oriented towards offering social and health services to the elderly, in which health professionals establish what is best for them according to their needs [7,8].

Although the current residential centers in Spain are far from the old asylums, they do not constitute actual residences. The reason for this is that they are developing a model focused on providing social and health services without considering that residents in nursing homes should be the axis around which all these services revolve.

It is, therefore, necessary to abandon this model and promote others that allow residents to become the central axis of all care activities. In other European countries, new autonomous residential models are being developed, thus allowing people to acquire a central role in their social and health care [72].

In northern Europe (Denmark and Sweden), as in North America, cohabitation units are the most widespread residential models. These co-housing units are apartments with approximately ten individual rooms where users share common areas (kitchen, gardens, living rooms). These apartments are adapted to the resident’s needs and degree of dependency. In addition, they receive 24 h care and health care if they have a high degree of dependency. These living units promote the residents’ autonomy and well-being, allowing them to feel at home and receive all the necessary social health care [73].

Another model in development in northern Europe is the so-called “housing” model, which makes it possible to provide home-based socio-health care to the elderly, since many do not want to leave their home and go to a residential center [74]. This residential model, implemented mainly in the United Kingdom, has some drawbacks since many of these people developed severe illnesses that cannot be cared for at home and they must move to a nursing home where they receive the required care [72]. It is also necessary to emphasize the importance of the architectural aspects of the residences. According to Parker et al. [65], the well-being of users of nursing homes depends, among many other factors, on how it was designed.

Thus, it is clear that the environment and design of nursing homes directly influence the residents. Furthermore, Knudstrup et al. [66] asserted that residential facilities function as a good workplace for the staff involved (doctors, nurses, and social-health care personnel) and ensure that residents have a dignified life. Knudstrup et al. [66], in a study conducted in Denmark, established a series of architectural aspects that should be present in nursing homes, as these elements promote a home-like environment.

These elements are the following: location of the nursing home, type of building, common areas (kitchens, dining rooms, living rooms, therapy room, and computer room), accessibility to the facility, interior design and furnishings (possibility of personalizing residents’ rooms), technologies (highlighting assistive technologies such as lifts, walkers, physical exercise equipment), colors and lights, and finally, outdoor spaces. They also recommended that small living units of 8–12 users have dining rooms, living and visiting rooms, pleasant decorations and furnishings, individual rooms, gardens, and outdoor spaces. Additionally, Takano et al. [75] indicated that users of residential centers prefer to live in residential homes close to urban centers to have contact with their family and friends. Other authors [76,77,78,79] emphasized the importance of nursing homes having green areas (gardens, terraces, balconies) since the contemplation of these areas stimulates the senses and establishes relationships and bonds of friendship among residents.

In Spain, the Basque Country developed a model called “Etxean ondo and day centers,” which aims to create living units that promote the autonomy and well-being of their residents [80], and in Soria (province of Castilla y León), several cohabitation units have already been created in the nursing home “Los Royales”.

We can also highlight the model proposed by Castilla y León, which aims to transform traditional nursing homes into cohabiting living units of about ten rooms. As for the common areas, the surface area of the kitchen-dining room should be about 20 m^2^ and that of the living rooms about 50 m^2^. In addition, all rooms must be single, no smaller than 17 m^2^, and should include a bathroom. Residents may personalize these rooms, and an extra bed may be requested for family members [81]. In addition, they will have spaces shared by several living units, such as visiting rooms, a gym, a computer room, a multipurpose room, a garden, and a library. The resident’s family is a fundamental element [81]. Therefore, family members can participate in the center’s activities with their relatives, even overnight. With this new model, Castilla y León aims to convert classic nursing homes into real homes, considering the preferences and wishes of the residents. This way, autonomy and self-determination are encouraged, and families can be part of the residents’ life projects.

In the future, it would be interesting to analyze and study the influence of variables not contemplated in the minimum requirements regulations, which depend exclusively on the center and could influence compliance in the elderly.

On the other hand, unfortunately, there were cases in which the elderly are victims of negligent care, abuse, and mistreatment [81]. One of the limitations of this work was that we did not control for the degree of compliance with the regulations in force in each autonomous community. In this sense, the agreement approved in 2022 by the Ministry of Social Rights and Agenda 2030 had very much in mind the control of the quality of residential centers and the homogenization of quality control standards. The dimensions to be evaluated will be articulated around the principles: (1) dignity and respect, (2) personalization and person-centered care, (3) participation, control, and choices, (4) right to health and personal well-being, and (5) proximity and community connections. Criteria related to the quality of the working conditions of the professionals in charge of providing support in the different services are also introduced as an element that impacts the axes mentioned above.

The model pursued in Spain is different from the European models mentioned above, since the size of nursing homes is not reduced, but smaller housing units are created within the existing residences. This commitment, which does not imply a dismantling of the nursing home sector as we know it, can facilitate the transition towards this model focused on the person, their needs, and preferences, with greater participation of the family that wants to continue participating in the life of the user. It is necessary to promote residential models focused on promoting the well-being and autonomy of residents, similar to European residential models, the most widespread being the creation of coexistence units. These coexistence units, implemented in countries such as Denmark and Sweden, allow the personal life project of each user to be the central axis of planning their social and health care.

Finally, it is vital to highlight the figure of healthcare professionals in nursing homes. A paradigm change occurs in health care, so professionals must accompany residents while respecting their preferences and identity. In this way, residents receive personalized and individualized care [81].

## 5. Conclusions

In conclusion, our study indicated that in Spain, each autonomous community has different regulations that generate inequality in providing facilities and/or qualified personnel in nursing homes. For all these reasons, we hope that the new framework established in the new agreement on uniform criteria for accreditation and quality of the centers and services of the System for Autonomy and Dependency Care can lay the foundations for (1) achieving regulatory homogenization nationally, with consequent reflection on more excellent uniformity in the characteristics of nursing homes, (2) reducing the growing inequality in the well-being of residents in different regions, and (3) ensuring that nursing homes create an environment that are as similar to home as possible.

The results of our estimations showed that the existence of requirements on the availability of physical and human resources was negatively associated with the price of the place (public or subsidized), while in the case of the coverage rate (public or subsidized), we only observed a positive association with the availability of physical resources being non-significant for human resources. The finding that the requirements on physical resources linked to common areas (dining room, visiting area, living rooms) showed a negative association with the price per place also lead us to assume that these requirements guarantee better habitability and cohabitation without implying a cost overrun.

On the other hand, the effect of GDP per capita only exhibited a positive, albeit minimal, relationship with the price per place but not with the coverage rate. This suggests that the economic inequalities between Spanish regions are not appreciably affecting the availability of places (public or subsidized), although their price does.

## Figures and Tables

**Table 1:** Regulations on the minimum requirements to be met by nursing homes in Spanish autonomous communities.

Autonomous Communities of Spain	Legal Regulations	References
Andalucía	Order of 28 July 2000, jointly issued by the Regional Ministries of the Presidency and Social Affairs, regulating the material and functional requirements of the Social Services and Social Service Centers of Andalusia and approving the application form for administrative authorizations.	[46]
Aragón	Decree 111/1992, of 26 May 1992, of the Diputación General de Aragón, regulates the minimum conditions that specialized social services and establishments meet.	[47]
Asturias	Resolution of 22 June 2009, of the Department of Social Welfare and Housing, which develops the criteria and conditions for the accreditation of social services and care centers in the Principality of Asturias.	[48]
Baleares	Decree 86/2010, of 25 June 2010, established the general principles and coordination guidelines for the authorization and accreditation of social services for the care of elderly and disabled persons and regulated the requirements for the authorization and accreditation of supra-insular residential services for these sectors of the population.	[49]
Canarias	Decree 63/2000, of 25 April, regulates the organization, authorization, registration, inspection, and regime of infractions and sanctions of centers for the elderly and their internal rules.	[50]
Cantabria	Order EMP/37/2010, of 18 March, uses the criteria and regulates the procedure for the accreditation of social service centers for the care of dependent persons.	[51]
Castilla-La Mancha	Order of 04/06/2013 of the Regional Ministry of Health and Social Affairs, which amends the Order of 21/05/2001 of the Regional Ministry of Social Welfare, regulates the minimum conditions of the centers intended for the elderly in Castilla-La Mancha.	[52]
Castilla y León	Decree 14/2001, of 18 January, regulates the conditions and requirements for the authorization and operation of centers of a social nature for the elderly.	[12]
Cataluña	Decree 205/2015, 15 September, on the regime of administrative authorization and prior communication of social services and the Registry of Entities, Services, and Social Establishments.	[53]
Comunidad Valenciana	Order of 4 February 2005, of the Department of Social Welfare, which regulates the system of authorization and operation of specialized social services centers for the care of the elderly.	[54]
Extremadura	Decree 298/2015, of 20November, approves the Regulation of authorization, accreditation, and Registration of Care Centers for the elderly in the Autonomous Community of Extremadura.	[55]
Galicia	Order of 13 April 2007, modifying that of April 18, 1996, which developed Decree 243/1995, of 28 July 1995, regarding the regulation of the specific conditions and requirements to be met by centers for the care of the elderly.	[56]
La Rioja	Decree 27/1998, of 6 March, regulating the categories and specific requirements of Residential Centers for the Elderly in La Rioja.	[57]
Madrid	Law 11/2002, of 18 December 2002, on the Regulation of the Activity of Social Action Centers and Services and the Improvement of Quality in the Provision of Social Services in the Community of Madrid.	[58]
Murcia	Decree 69/2005, of June 3, established the minimum conditions to be met by publicly or privately owned residential centers for the elderly.	[59]
Navarra	Regional Decree 209/1991, of 23 May, developing Regional Law 9/1990, of November 13, on the system of authorizations, infractions, and penalties in Social Services matters.	[60]
Basque Country	Order of 4 February 2005, of the Department of Social Welfare, regulating the authorization and operation of specialized social services centers to care for the elderly.	[61]

**Table 2:** Variables analyzed in the regulations for nursing homes in Spain.

Dining rooms (required minimum area)
Kitchens (required minimum area)
Living room (required minimum area)
Visiting room (availability and required minimum area)
Chapel and/or mortuary (availability)
Outdoor recreation area (availability)
Room customization
Hairdressing area (availability)
Lift (number and availability)
Sanitary mobilization cranes (availability)
Sociocultural activities program
Doctors ratio * (healthcare staff)
Nurses ratio * (healthcare staff)

* mean doctor and nurses to resident ratio.

**Table 3:** Variables analyzed in the regulations of the different autonomous communities of Spain.

	AN	AR	AS	BA	CAN	CANT	CLM	CyL	CAT	C.VAL	EXT	GAL	MAD	MUR	NAV	P.VAS	RIOJA
Dining room dimension	2 m^2^ per user min. 15 m^2^	-	2 m^2^ per user	2 m^2^ per user min. 30 m^2^	2 m^2^ per user min. 15 m^2^	2 m^2^ per user	≤ 45 places-30 m^2^ 46–80 places -40 m^2^ More than 80 places- 80 m^2^	-	-	-	-	2 m^2^ per user min. 30 m^2^	1 m^2^ per user a dimension not less than 10 m^2^	-	-	-	-
Kitchen dimensions	15 m^2^	-	12 m^2^	≤25 places- 5 m^2^ ˃25 places 12.5 m^2^	10 m^2^, increasing 0.5 m^2^ for every 30 users	12 m^2^	20 m^2^	10 m^2^	min. 8 m^2^ max. 50 m^2^	19 m^2^	-	-	-	-	-	-	≤40 places greater than 12 m^2^ ˃40 places more than 20 m^2^
Living room dimensions	min. 30 m^2^ 2 m^2^ per user	1.8 m^2^ per user	min. 15 m^2^ 2 m^2^ per user	min. 30 m^2^ 1.8 m^2^ per user	min 30 m^2^	min 30 m^2^ 1.50 m^2^ per user	min. 25 m^2^. If there are ≤45 places, 2 living rooms; if there are between 46–80 places, at least 3 living rooms and if there are more than 80 places, at least 4 living rooms	min 30 m^2^ (5.60 m^2^ per user	More than 25 places, at least 2 living rooms	-	1.8 m^2^ per user; if there are less than 15 users, there must be a 30 m^2^ room	min. 30 m^2^ 1.80 m^2^ per user)	minimum 12 m^2^ (1.80 m^2^ per user)	-	2 m^2^ per user. If there are less than 25 seats, the room must have a minimum of 12 m^2^	-	minimum 20 m^2^
Visiting room	min.15 m^2^ There will be one room for every 60 users	-	-	-	-	In all residential centers, there must be a visiting room	All residences will have at least one visiting room	-	-	-	-	-	-	They will have a minimum area of 12 m^2^	-	-	-
Wake chapel/Mortuary	More than 60 places—at least one room for a coffin	There will be a room if there is no funeral home in the municipality	-	-	More than 60 places; if there is no funeral home in the municipality, there will be a room in the residences	More than 50 places; if there is no funeral home in the municipality, there will be a room	There will be a wake room if there is no funeral home in the municipality	-	-	-	There will be a room if there is no funeral home in the municipality	If the residence has more than 60 places, there will be a room	In centers with more than 100 places	It is mandatory to have a room in municipalities where there are no funeral homes	-	In residences with more than 70 places	wake room of at least 10 m^2^
Outdoor recreation area	if it is not may, these areas inside of the home must situate in the center, less than 50 m from the parks and gardens public.	-	-	The center’s residential must count with landscaped zones. The center residential must situate near gardens/parks public.	Shall count on spaces exterior landscaped whenever possible	-	The residences shall count on gardens in their interior	-	-	The zones will have one surface minimum of 3 m^2^ per username	-	-	--	-	-	-	-
Room customization	-	-	It is possible	It is possible	-	-	It is possible	-	It is possible	-	-	It is possible	-	-	-	-	-
Hairdressing areas	-	-	-	-	-	In centers ˃50 places, there will be a space dedicated to the hairdresser	This service will be available in centers with more than 80 beds.	-	-	The creation of an area dedicated to the hairdresser is contemplated	-	-	-	-	-	-	-
Lifts	minimum 1 lift for every 60 users	-	-	Minimum 1, increasing to 1 per every 65 users	-	-	2 elevators in residences with more than 45 beds	-	-	-	-	--	-	Minimum 1 lift	-	-	Minimum 1 elevator if there are more than 40 places
People mobilization cranes	Enough number for people who needs them	-	-	-	-	-	-	-	Enough number for people who needs them	Enough number for people who needs them	-	-	-	-	-	-	-
Program of sociocultural activities	-	-	-	-	-	-	-	-	-	All residential centers must have an annual program of sociocultural activities.	-	-	-	Residential centers must have an annual program of sociocultural activities	-	All residential centers must have an annual program of sociocultural activities.	-

(-): no data. Min. Mínimum. Max. Maximum. AN: Andalucía, AS: Asturias, BA: Baleares, CAN: Canarias, CANT: Cantabria, CLM: Castilla-La Mancha, CyL: Castilla y León, C.VAL: Comunidad Valenciana, EXT: Extremadura, GAL: Galicia, MAD: Madrid, MUR: Murcia, NAV: Navarra, P.VASCO: Basque Country.

**Table 4:** Analysis of the ratio of doctors and nurses in the regulations of the different autonomous communities of Spain.

	AN	AR	AS	BA	CAN	CANT	CLM	CyL	CAT	C.VAL	EXT	GAL	MAD	MUR	NAV	P.VAS	RIOJA
Doctor ratio	-	-	-	-	-	-	Residences with more than 45 beds with the physical presence of a doctor 5 h a week, which will gradually increase by 1 h per week for every ten residents or fraction, up to residences with 119 beds. Those with 120 beds or more with 17 weekly hours of the physical presence of a doctor, increasing by eight weekly hours for every 20 users or fraction.	Residences with up to 25 users will require a minimum service of 1 h per day. Additionally, for every 25 users or fraction, a service of one more hour per day will be made.	-	At least 3 h per week for every ten places or a fraction in centers with less than 100 places. In centers with more than 100 places, 10 h per week for every 25 places or a fraction. The minimum weekly benefit will be five hours.	One doctor	In centers with fewer than 40 assisted places, the localized presence of a doctor must be available 24 h a day.	-	In residences with up to 40 users, a minimum service of one hour per day will be required. In addition, for every 20 more users, the doctor will provide a service of one more hour per day.	-	Establishes the ratio of doctors based on the degree of dependency that the residents of their residential centers have.	It establishes the ratio of doctors based on the level of occupancy of its residential centers. Level 1: there will be a doctor 20 h/per week Level 2: there will be a doctor 40 h/per week Level 3: there will be a doctor 60 h/week Level 4: there will be a doctor 80 h/week
Nursing ratio	-	-	The ratio of 0.025 per user in a situation of degree II dependency and 0.035 per user in a situation of degree III dependencies.	-	-	-	Mini-residences with 20 weekly hours of the physical presence of a nurse. Medium-sized residences with 40 weekly hours of the physical presence of a nurse. Large residences with more than 80 beds with 40 weekly hours of the physical presence of a nurse will increase by 20 weekly hours for every 40 users or fraction.	Up to 25 users will require a minimum service of 2 h daily. One hour per day will also be provided for every eight users or a fraction.	-	Minimum of 8 h per week for every ten places or fraction proportionally. In a center with a high dependency unit: nurses with physical presence 24 h a day.	One nurse for every 25 residents	In centers with fewer than 40 assisted places, there must be a localized presence of a doctor and a nurse 24 h a day. Those with a higher number of assisted places, in addition to the localized presence of medical personnel, must have the physical presence of a nurse 24 h a day. - A nurse with a minimum service of 1 h daily for up to 40 users. Additionally, every 20 users or fraction will be provided for one more hour a day. A graduate in nursing with a permanent presence in the center. Nursing: Grade 0 and I= 0.027, Grade II and II= 0.032. You will have a nurse in the following proportion: Level 1: 60 h/week. Level 2: 80 h/week. Level 3: 100 h/week. Level 4: 160 h/week.	-	A nurse with a minimum service of 1 h daily for up to 40 users. Additionally, every 20 users or fraction will be provided for one more hour a day.	A graduate in nursing with a permanent presence in the center.	Nursing: Grade 0 and I = 0.027, Grade II and II = 0.032.	A nurse in the following proportion: Level 1: 60 h/week. Level 2: 80 h/week. Level 3: 100 h/week. Level 4: 160 h/week.

(-): no data. Min. Mínimum. Max. Maximum. AN: Andalucía, AS: Asturias, BA: Baleares, CAN: Canarias, CANT: Cantabria, CLM: Castilla-La Mancha, CyL: Castilla y León, C.VAL: Comunidad Valenciana, EXT: Extremadura, GAL: Galicia, MAD: Madrid, MUR: Murcia, NAV: Navarra, P.VASCO: Basque Country.

**Table 5:** Prices and coverage rate for nursing homes and regional GDP per capita. 2020.

	Price (EUR /Year)		Coverage Rate			Requirements		GDP Per Capita
	Public Places	Subsid. Places	Public Places	Subsid. Places	Total	Facilities	Human Resources	
Andalucía	18,000.00	20,098.32	1.96	1.02	2.98	8	0	17,545
Aragón	19,461.52	19,461.52	3.91	2.70	6.61	2	0	26,642
Asturias	16,349.15	16,349.15	2.99	2.63	5.63	5	1	20,947
Baleares	24,022.84	23,798.37	1.95	0.99	2.94	6	0	21,551
Canarias	25,334.93	30,189.22	1.15	1.62	2.76	5	0	17,199
Cantabria	19,767.76	19,767.76	3.26	1.44	4.70	6	0	22,048
Castilla y León	15,980.59	15,980.59	5.91	1.95	7.86	4	2	22,925
Castilla La Mancha	16,365.66	16,049.74	4.93	1.99	6.92	11	2	19,257
Cataluña	20,414.34	20,414.34	3.10	1.25	4.35	4	0	27,745
C. Valenciana	21,960.00	21,960.00	1.80	0.90	2.70	7	2	20,780
Extremadura	15,591.57	15,591.57	3.79	2.91	6.71	4	2	17,834
Galicia	18,000.00	18,252.00	1.83	1.10	2.94	6	2	21,730
Madrid	19,000.00	20,407.15	1.86	2.50	4.36	3	0	32,332
Murcia	19,200.00	20,987.50	1.42	0.90	2.33	6	2	19,692
Navarra	13,349.07	22,136.43	2.24	2.18	4.42	2	1	28,822
Basque Country	27,342.67	34,721.45	2.79	1.02	3.81	4	2	30,338
La Rioja	10,423.57	22,936.08	2.52	2.38	4.90	6	2	25,666

GDP, gross domestic product. Own work using public and subsidized prices and coverage rates from Servicios sociales dirigidos a personas mayores en España. Diciembre 2020—Instituto de Mayores y Servicios Sociales (imserso.es) and GDP per capita (GDPpc) from Spanish Regional Accounts (National Institute of Statistics; INEbase/Economía/Cuentas económicas/Contabilidad regional de España/Últimos datos). Requirements for facilities: sum of binary variables defined for each one of requirements related to dining rooms (required minimum area), kitchens (required minimum area), living room (required minimum area), hairdressing area (availability), visiting room (availability and required minimum area), chapel and/or mortuary (availability), sanitary mobilization cranes (availability), lift (number and availability), room customization, outdoor recreation area (availability), sociocultural activities program. Requirements for human resources: sum of binary variables defined for requirements related to the ratio of nurses and doctors. Coverage rate: number of beds placed per 100 persons aged 65 and over.

**Table 6:** Correlation matrix.

	Price (Public Place)	Coverage Rate (Public Price)	Price (Subs. Place)	Coverage Rate (Subs. Place)	GDP Per Capita	No. Req. Related to Facilities	No. Req. Related to Human Resources
Price (public place)	1.000						
Coverage rate (public price)	−0.3264	1.000					
Price (subs. place)	0.6685	−0.4964	1.000				
Coverage rate (subs. place)	−0.5768	0.4275	−0.4164	1.000			
GDP per capita	0.0064 *	0.0199	0.2817	0.1863	1.000		
No. requirements related to facilities	−0.0421 ***	0.1760 *	−0.1746 **	0.4092 *	−0.4610	1.000	
No. requirements related to human resources	−0.3046 ***	0.2541	−0.1136 **	0.0094	−0.1385	0.2550	1.000

***, ** and * denote statistical correlation at the 1%, 5%, and 10% level. GDP, gross domestic product. Requirements for facilities: dining rooms (required minimum area), kitchens (required minimum area), living room (required minimum area), hairdressing area (availability), visiting room (availability and required minimum area), chapel and/or mortuary (availability), sanitary mobilization cranes (availability), lift (number and availability), room customization, outdoor recreation area (availability), and sociocultural activities programs. Requirements for human resources: ratio of nurses and ratio of doctors.

**Table 7:** OLS regression analysis for public price and subsidized price.

	Public Places. Price (Thousand EUR/Year)				Subsidized Place. Price (Thousand EUR/Year)			
	R1	R2	R3	R4	R1	R2	R3	R4
Dining rooms	−1.52 **				−0.78 **			
	(0.48)				(0.35)			
Kitchens	−7.42 **				−2.02 **			
	(1.48)				(0.45)			
Living room	−9.85 **				−11.50 **			
	(1.64)				(2.90)			
Hairdressing area	2.05				5.64 **			
	(1.87)				(1.60)			
Visiting room	−4.80 **				−12.10 **			
	(1.01)				(5.02)			
Chapel and/or mortuary	8.19 **				3.40			
	(1.81)				(5.43)			
Sanitary mobilization cranes	−0.51				−3.97			
	(1.65)				(4.94)			
Lift	4.19 **				9.90 **			
	(1.78)				(2.34)			
Room customization	4.41 **				7.71			
	(1.44)				(4.31)			
Outdoor recreation area	2.94				13.46			
	(1.72)				(8.15)			
Sociocultural activities program	−1.50 *				−4.80 **			
	(0.34)				(1.75)			
Ratio of doctors	−2.35 *				−5.88 *			
	(1.10)				(2.45)			
Ratio of nurses	−7.41 *				−3.33 *			
	(1.93)				(1.50)			
Requirements for facilities		−3.05 **	−3.14 **	−3.15 **		−3.09 **	−2.92 **	−2.60 **
		(0.58)	(0.50)	(0.59)		(0.69)	(0.59)	(0.65)
Requirements for human resources		−4.38 **	−4.11 **	−4.11 **		−2.35 **	−2.08 **	2.05 **
		(1.20)	(1.18)	(1.23)		(0.42)	(0.23)	(0.24)
GDP per capita		0.32 **		0.31 **		0.27		0.27
		(0.07)		(0.07)		(0.32)		(0.28)
Coverage ratio (public or subs.)			−0.91	−0.92			−1.10	−1.10
			(0.88)	(0.92)			(1.77)	(1.77)
Constant	29.96 **	20.58 *	21.88 **	21.57 *	28.89 **	15.67	33.13 **	25.44 *
	(2.08)	(8.20)	(3.36)	(8.26)	(6.23)	(9.69)	(5.17)	(9.40)
N	17.00	17.00	17.00	17.00	17.00	17.00	17.00	17.00
R2	0.90	0.79	0.76	0.76	0.71	0.79	0.72	0.77
F-test	27.16	30.45	30.85	29.59	31.82	32.41	32.01	31.74
*p*-value	0.00	0.00	0.00	0.00	0.00	0.00	0.00	0.00

N, number of observations; R^2^: goodness of fit statistic; F: F-test statistic tests whether any independent variables in a multiple linear regression model are significant. GDP; gross domestic product. In this table, we report the estimated coefficients from OLS regressions. Standard errors between parentheses. * *p* < 0.05; ** *p* < 0.001. *p*-values below 0.05 were considered significant.

**Table 8:** OLS regression analysis for a rate of coverage for public places and subsidized places.

	Public Places. Price (Thousand EUR/Year)				Subsidized Place. Price (Thousand EUR/Year)			
	R1	R2	R3	R4	R1	R2	R3	R4
Dining rooms	−2.64 **				−0.30			
	(1.03)				(0.99)			
Kitchens	2.20				−0.09			
	(2.19)				(1.12)			
Living room	1.71 **				3.20 **			
	(0.54)				(1.05)			
Hairdressing area	2.20 **				3.22 **			
	(1.05)				(1.17)			
Visiting room	3.19 **				4.18 **			
	(1.80)				(1.43)			
Chapel and/or mortuary	2.61				0.46			
	(2.05)				(1.05)			
Sanitary mobilization cranes	0.89 **				−0.80			
	(0.28)				(0.91)			
Lift	21.81				−0.67			
	(2.30)				(0.66)			
Room customization	−0.54				−0.22			
	(2.02)				(1.03)			
Outdoor recreation area	2.08 **				0.87 **			
	(0.40)				(0.23)			
Sociocultural activities program	1.82				1.19 **			
	(1.31)				(0.47)			
Ratio of doctors	0.30				0.97			
	(0.81)				(0.78)			
Ratio of nurses	−1.27				−1.83			
	(3.09)				(1.58)			
Requirements for facilities		0.60 *	0.57 *	0.61 *		0.14 *	0.16 *	0.15 *
		(0.28)	(025)	(0.28)		(0.07)	(0.07)	(0.07)
Requirements for human resources		0.20	0.18	0.18		0.07	0.05	0.05
		(0.36)	(2.37)	(2.38)		(0.19)	(0.16)	(0.17)
GDP per capita		0.04		0.03		−0.00		0.02
		(0.08)		(0.08)		(0.04)		(0.04)
Public price (public or subs.)			−0.08	−0.08			−0.07 *	−0.08 *
			(0.08)	(0.08)			(0.03)	(0.03)
Constant	4.62 **	4.08 **	3.79 **	3.79 **	2.29 *	2.39 *	4.05 **	3.56 *
	(1.00)	(1.47)	(1.32)	(1.01)	(1.02)	(1.01)	(0.82)	(1.25)
N	17.00	17.00	17.00	17.00	17.00	17.00	17.00	17.00
R2	0.68	0.69	0.65	0.66	0.74	0.78	0.72	0.73
F-test	25.72	25.74	25.76	25.78	21.83	22.83	22.81	22.26
*p*-value	0.00	0.00	0.00	0.00	0.00	0.00	0.00	0.00

N, number of observations; R^2^: goodness of fit statistic; F: F-test statistic tests whether any independent variables in a multiple linear regression model are significant. GDP, gross domestic product. In this table, we report the estimated coefficients from OLS regressions. Standard errors between parentheses; *p < 0.05; ** *p* < 0.001. *p*-values below 0.05 were considered significant.
